# Assessing the Potential of Robotics Technology for Enhancing Educational for Children with Autism Spectrum Disorder

**DOI:** 10.3390/bs13070598

**Published:** 2023-07-16

**Authors:** Maha Alghamdi, Noura Alhakbani, Abeer Al-Nafjan

**Affiliations:** 1 Information Technology Department, College of Computer and Information Sciences, King Saud University, Riyadh 11543, Saudi Arabia; 443204753@student.ksu.edu.sa (M.A.); nhakbani@ksu.edu.sa (N.A.); 2 Computer Science Department, College of Computer and Information Sciences, Imam Mohammad Ibn Saud Islamic University (IMSIU), Riyadh 11432, Saudi Arabia

**Keywords:** robotics, education, autism spectrum disorder, systematic literature review

## Abstract

Robotics technology has been increasingly used as an educational and intervention tool for children with autism spectrum disorder (ASD). However, there remain research issues and challenges that need to be addressed to fully realize the potential benefits of robot-assisted therapy. This systematic review categorizes and summarizes the literature related to robot educational/training interventions and provides a conceptual framework for collecting and classifying these articles. The challenges identified in this review are classified into four levels: robot-level, algorithm-level, experimental-research-level, and application-level challenges. The review highlights possible future research directions and offers crucial insights for researchers interested in using robots in therapy. The most relevant findings suggest that robot-assisted therapy has the potential to improve social interaction, communication, and emotional regulation skills in children with ASD. Addressing these challenges and seeking new research avenues will be critical to advancing the field of robot-assisted therapy and improving outcomes for children with ASD. This study serves as a roadmap for future research in this important area.

## 1. Introduction

Today, advances in artificial intelligence and technology have facilitated the development of more advanced and capable robots. Robotics has been applied across a wide range of medical and non-medical fields, resulting in the development of numerous types and approaches that target different application areas. These applications are used for both healthy individuals and those with medical conditions, and they all share a common research approach: designing and developing robots to perform specific tasks or behaviors and conducting experiments to evaluate their effectiveness.

Autism spectrum disorders (ASDs) are considered one of the serious disabilities, according to the *Diagnostic and Statistical Manual of Mental Disorders* [1]. Children with ASD have functional impairment in their social interactions and communication across various contexts and present restricted, repetitive patterns of behavior, such as stereotyped or repetitive motor movements or commitment to routines [2]. They have difficulty recognizing body language, making eye contact, explaining personal feelings, and understanding the emotions of other people. Given these limitations, health and educational professionals frequently struggle to motivate and engage children with ASD in treatment and learning activities. Participation in a diverse range of social, play, educational, and therapeutic activities is essential for acquiring knowledge that is necessary for cognitive and social development [2].

Traditional intervention approaches usually necessitate intensive support under the direct supervision of well-trained professionals. However, professional care and amenities are not constantly available to many autistic individuals because of high intervention costs and/or a lack of available qualified therapists [3].

Several interventions for children with ASD have been developed with the goals of improving cognitive ability and daily living skills, increasing their ability to interact and engage in the community, and trying to reduce symptoms. During therapy sessions, for example, assistive technologies have been used. This intervention is driven by the societal need for technological innovations that can support and improve current therapies for the increasing number of children with autism [4].

Recently, robots have been used in a variety of assistive scenarios, such as meeting various human needs and assisting in the rehabilitation of individuals with ASD [5]. The clinical use of social or interactive robots appears to be promising for improving the social skills of children with ASD. Humanoid robot-assisted teaching and intervention programs for children with ASD are rapidly evolving [6].

This study offers a novel contribution to the existing literature on robot-assisted therapy for children with ASD by proposing a comprehensive classification scheme for the challenges and issues in Robot-Based Interventions for Therapeutic Educational Support in Autism. The primary goals of this review are to categorize and summarize the literature related to robot educational/training interventions, as well as provide a conceptual framework for collecting and classifying these articles. The classification scheme will be helpful for studies of the literature on the use of robots in training and education for children with ASD.

Moreover, through a thorough review of the literature and collection of challenges, the study identified and classified issues into four levels: robot-level, algorithm-level, experimental-research-level, and application-level issues. The proposed classification scheme provides a holistic framework for researchers and practitioners to identify key challenges and issues across different levels of intervention. Moreover, the study highlights possible future research directions to address these challenges and issues, providing a roadmap for future research in the field of robot-assisted therapy for children with ASD. Overall, this study offers a unique and valuable contribution to the field, emphasizing the importance of addressing challenges and seeking new research avenues to fully achieve the potential benefits of robot-assisted therapy.

The research questions that guided this work are as follows: what are the research issues and challenges that need to be addressed by researchers in the field of robot education and training for children with ASD, and how can these issues be addressed to fully achieve the potential benefits of robot-assisted therapy?

In the following sections, we start by illustrating the methodology we use for this systematic review. Section 2 is an outline of the research methodology. Section 3 details the proposed classification framework for the literature review, and Section 4 presents the discussion. Section 5 provides insights for future research. Finally, Section 6 presents the conclusions.

## 2. Research Methodology

Articles on using robots in education and training for children with ASD are scattered across conference proceedings and journals with different areas of improvement, such as the development of communication and social skills and improved verbal/non-verbal expressions, imitation skills, and cognitive skills. This section describes the method used for finding the relevant articles, along with the article-selection criteria and filtering processes.

Although robot-based intervention for autism education and therapy research is a relatively new and emerging topic, it has attracted researchers from different disciplines and has gained momentum as a field of academic research. The results of studies on this subject have been published in scientific journals and conferences across the major bibliometric databases, including Google Scholar, Scopus, and the Web of Science (WoS). 

**Figure 1:**
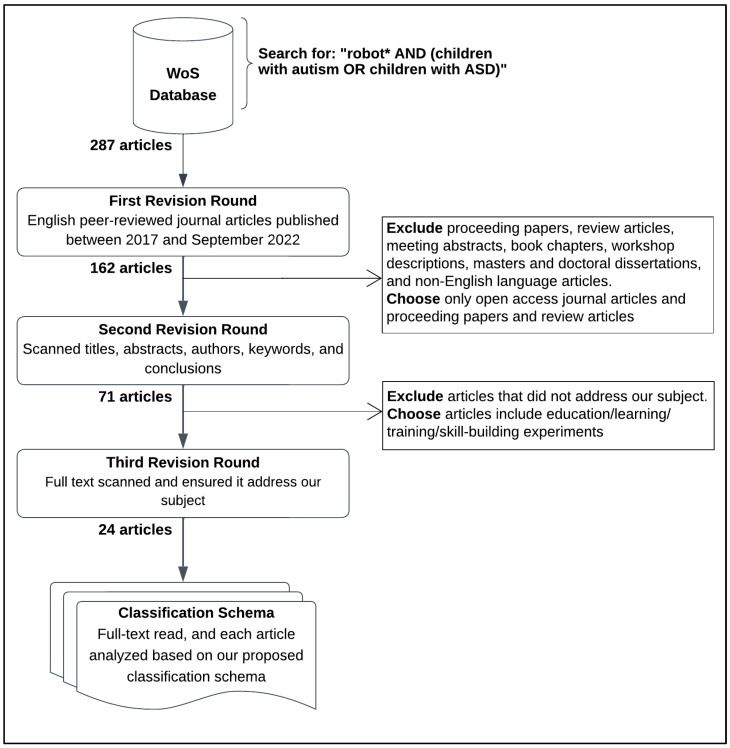
Article-filtering process.

We searched the WoS [7] to obtain a comprehensive bibliography of the academic literature on using robots with ASD children in the education and skill-building field. The WoS database provides the most trusted, world-leading citations from databases such as Science Direct (Elsevier), IEEE/IEE Library, ACM Digital Library, and Springer Link Online Libraries. Specifically, we searched the WoS Core Collection database for recent articles from 2017 to September 2022. Using the search bar, we input the phrase “robot* AND (children with autism OR children with ASD)” to include all word forms, such as “robot”, “robots”, and “robotics”, and where robots are employed in autism therapy for children. Figure 1 shows the search results, along with the filtering process.

The initial search resulted in 287 research papers. From these, we selected journal articles and excluded proceeding papers, review articles, meeting abstracts, book chapters, workshop descriptions, masters and doctoral dissertations, and non-English-language articles. The first revision cycle ended with 162 search results. Then, we carried out a second screening procedure in which we manually reviewed each research paper to exclude articles that did not address the subject.

In the second revision round, we scanned article titles, abstracts, authors, keywords, and conclusions. Then, we filtered the resulting papers by selecting only the ones related to the education and skill-building field. We left with 71 articles.

Finally, in the third revision cycle, we read the full text for the remaining 71 articles, and we eliminated 47 more articles because their topics were irrelevant to our theme. The remaining 24 articles were examined according to our classification schema.

## 3. Classification Method

**Figure 2:**
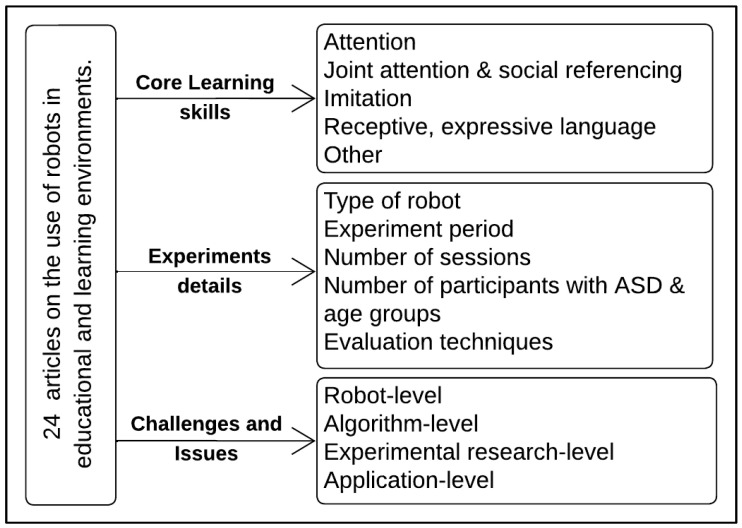
Classification scheme.

To systematically explore and examine the research findings on the use of robots in education and training for children with ASD, an approach for classifying the literature was produced. This classification scheme was developed by categorizing the studies of the 24 articles that remained after the filtering step. Figure 2 shows a representation of the classification scheme, which depends on core learning skills introduced by [8] and the experiment details.

Some abilities are targeted to compensate for deficiencies shared by many children on the autism spectrum. These are core skills that are considered to be building blocks and essential prerequisite tools that allow the children to learn faster. Owing to the significant impact they have on later development, these skills are considered crucial to the success of any educational/training intervention [8]. Whitman and DeWitt, in [8], provide a blueprint for an educational intervention program for children with autism. There are nine core skills: attention, joint attention, social referencing, imitation, receptive, expressive language, matching, sequencing, and attachment/bonding. We chose the most common skills between the extracted studies.

## 4. Results

### 4.1. Article Classification by Targeted Learning Skills

Numerous studies use robots to aid in the growth and development of children with ASD by combining therapy and education [4]. Many interventions aim to address deficiencies and delays and develop competencies in the following core learning skills: attention, joint attention, social referencing, imitation, and receptive and expressive language. These core learning skills are especially important in the initial stages of intervention programs for children with ASD [8].

Table 1 summarizes the learning skills, in addition to the following points that describe the core skills included in the classification. In our review, we found that 25% (6 articles) targeted joint attention and social referencing in their study; 16.6% (4 articles) target imitation skills; 8.3% (2 studies) focused on receptive and expressive language, with 1 article focused on attention skills; and 54% (13 articles) focused on different skills, such as emotional expression, verbal, non-verbal communication, and general social skills, which are not included in the core learning skills defined by [8]. Finally, one study did not identify the targeted skills. 

#### 4.1.1. Attention

This classification includes articles focusing on attention skills and how to improve them. For example, Mehmood et al. in [9] described the impact of robotic stimuli on the attentional behavior of children with ASD. The robotic stimuli were classified into three types: visual, speech, and motion. For the visual social stimulus, the robot uses RGB light to change the color of its eyes; for the speech social stimulus, the robot greets the participant by saying, “Hello! Nice to meet you”. In addition, for the motion social stimulus, it stands and waves its right hand. Their results show that the speech stimulus captures children’s attention faster than visual and motion stimuli can in terms of latency in shifting attention and the number of attentions paid.

**Table 1:** Article classification by targeted learning skill.

Targeted Learning Skill	Explanation	References
Attention	The development of more sophisticated cognitive functions requires attention. It is extremely typical for children with ASD to struggle with attention.	[9]
Joint attention and social referencing	Children with autism are unlikely to participate in a shared focus on something (e.g., other people, objects, or events) with another person, for example, shift their gaze to someone who is reading to them. Joint attention is a behavior in which two people focus on the same object or event.	[10,11,12,13,14,15]
Imitation	Infancy and childhood learning are shaped primarily by imitation, which also has an impact on play, social interaction, communication abilities, and knowledge acquisition, including socioemotional understanding.	[11,14,16,17]
Receptive and expressive language	Language deficiencies are a core diagnostic characteristic of autism, which is not surprising given their problems in preverbal communication (e.g., limited use of social gestures), leading to difficulties in expressing and understanding words correctly.	[12,18]
Other	This includes emotional expression, verbal and non-verbal communication, general social skills, and so forth.	[19,20,21,22,23,24,25,26,27,28,29,30,31]
Undefined	Studies have not identified or mentioned the targeted skills to be improved.	[32]

#### 4.1.2. Joint Attention and Social Referencing

This classification includes articles targeting joint attention and social referencing. For instance, Kumazaki et al., in [10], compared the behavior of children with ASD to that of children with typical development during a joint-attention-elicitation task in which the children interacted with either a human or a robotic agent. The agent attempted to induce joint attention (JA) by gazing alternately towards the child and images on the left and right sides for specific durations. The task was repeated four times during each interaction session, with videos recorded for offline analysis. The results show that robotic intervention improves joint attention in children with ASD more than human-agent intervention does. After interacting with the robot, the children performed better in the joint-attention task with humans.

In addition, Ilijoski et al. in [14] overcome a lack of equipment and personnel by extending the concept of robot therapy using a web and mobile application. The robot is placed in front of the child and controlled by a therapist via a remote keyboard or computer during the therapy. An interaction between the child and the robot is thus established. The therapist guides the session with a variety of exercises. In this way, different therapeutic and educational exercises and games, such as turn-taking, joint attention, and collaborative games, can be used. 

Ali et al. in [13] also conducted a multi-robot-based therapy that aims to improve the social interaction skills of children with ASD. Multi-robot interventions enable ASD children to become acquainted with multi-human communication. The triad human communication scenario focused on the parameters of an ASD child’s joint attention, command following, and response. In this study, NAO robots stood 1 m away from the child. The robots had auditory, visual, and combination commands for their interaction with the child, including speech, sitting, standing, and waving gestures. Each command was repeated three times by each robot. The total time was 15 min. The findings show that the social communication skills of children with ASD improved by 86% overall.

#### 4.1.3. Imitation

This classification includes articles targeting different forms of imitation skills. For example, Conti et al. in [11] presented an empirical evaluation of robot-assisted imitation training. The task focused on teaching gross motor imitation skills to children, where the child learned to use certain motor skills in repeated patterns within the same sequence. The findings demonstrate that a robot assistant could be successfully integrated into the standard treatment of autistic children with mild, moderate, and severe intellectual disability. Following the robot training, the children were actually able to perform new gross motor imitations completely.

In addition, Telisheva et al., in [17], performed a series of different quantitative analyses to present the experiences of an ASD child with robots while engaging in play-based activities. The researchers created a variety of robot-performed tasks. Overall, there were seven action blocks (“Songs”, “Dances”, “Emotions”, “Touch me”, “Storytelling”, “Imitation”, and “Social Acts”) with a total of 26 sub-activities in two languages (Kazakh and Russian). These multipurpose activities integrate tasks involving imitation, turn-taking, emotions, and other social and behavioral skills to practice social and emotional skills. The findings indicate that robots can interact socially with children and influence their social behaviors over time.

#### 4.1.4. Receptive and Expressive Language

This classification includes articles focusing on receptive and expressive language. For example, Huijnen et al., in [12], aimed to gain insight into as many different roles as possible for robots in ASD interventions. One of these roles is that of a trainer, who is responsible for a wide range of skills, such as following up on instructions, which is a way to improve receptive and expressive language. The result of the experiment anticipates that robots will become valuable tools in the hands of professionals.

#### 4.1.5. Other

This classification includes articles focusing on other skills, such as emotional expression, verbal and non-verbal communication, and general social skills. For example, in [29], the authors investigated the impact of a humanoid robot on the development of socioemotional skills in children with ASD. The performance of the children’s in-game scenarios that were aimed at developing facial-expression-recognition skills was presented. The main findings of this study support that a humanoid robot is a useful tool for developing socioemotional skills in the intervention of children with ASD, owing to the observed engagement and positive learning outcomes.

Furthermore, the study in [21] focused on two important types of social skills: distrust and deception. The purpose of this study was to determine whether children with ASD could learn these skills from robot–child interactions by developing an experimental setup for children with ASD. The emergence of distrust and deception behaviors in children is a significant milestone in their social–cognitive development and has a profound impact on their real life. The experimenter invited the children to play a hide-and-seek game with the robot to find tokens. This study confirms that social robots can help children with ASD learn some social rules and demonstrates that children’s perception of the robot is important in their social learning.

The authors in [26] described the child–robot interaction scenarios created as part of the European BabyRobot project to help children with autism explore elements crucial in developing their visual-perspective-taking (VPT) skill, that is, the ability to see the world through the eyes of another person, taking into account what they see and how they see it. The study likewise presented the standard pre- and post-assessments that were conducted with the children to assess their progress. The robot was used to implement the games. This study was also the first attempt to improve the VPT skills of autistic children through their interaction with a humanoid robot.

#### 4.1.6. Undefined

This classification includes studies that did not identify or mention the target skills to be improved. For example, in [32], the aim of the study was to discover how to incorporate robots into current education and therapy interventions for children with ASD. Focus groups and co-creation sessions were conducted in this qualitative study to elicit the requirements for robot-assisted interventions. This research produced insights on robots, end users, the environment, and practical-implementation requirements, as well as a template to systematically describe robot interventions with new approaches.

### 4.2. Article Classification by Experiment Details

All 24 papers extracted after applying the research methodology and filtering were experimental studies. We classified these papers further according to the experiment details below.

#### 4.2.1. Type of Robot

Recently, robots have been used in a variety of assistive scenarios, such as meeting various human needs and assisting in the rehabilitation of individuals with ASD [5]. The clinical use of social or interactive robots appears to be promising for improving the social skills of children with ASD. Humanoid robot-assisted teaching and intervention programs for children with ASD are rapidly evolving [6].

Table 2, below, summarizes the article classification by robot type. We found that 41% (10 articles) used a NAO robot to carry out the experiment, 20% (5 articles) used a Kaspar robot, and 37.5% (9 articles) used different types of robots to run the experiment, such as the CommU robot, Maria T21, and LEGO robot.

NAO is a humanoid robot merchandised by SoftBank Group Corporation. He has 25 degrees of freedom, which allows him to perform most human body movements. NAO microphones have a sensitivity of 20 mV/Pa +/−3 dB at 1 kHz and an input frequency range of 150 Hz–12 kHz, according to official Aldebaran manufacturer documentation [16].

A novel music-based scenario using a NAO robot was designed by [25] to teach music principles to children with autism, while also enhancing their social and cognitive abilities through engaging in musical activities. The findings show that the NAO robot can teach musical notes and rhythms to individuals with autism when used as a tool and assistant. Furthermore, significant improvements in social/cognitive skills and a positive effect of this program on fine-motor imitation skills were observed.

Kaspar is developed at the Adaptive Systems Research Group at the University of Hertfordshire (UK). It is a semi-autonomous humanoid robot the size of a sitting infant or toddler. It makes motions and (limited) facial expressions by moving the head, chest, arms, and hands. Furthermore, sound and speech can be used as further interaction settings. Each arm has 3 degrees of freedom, and various parts of its face (for example, the eyes, mouth, and eye lids) have distinct motors that can be activated. Kaspar is seated on a table and is unable to stand or walk away (its legs are not activated). Kaspar may be operated by activating sensors in various body parts (hands, belly, feet, and head), as well as with a preprogrammed remote control [12].

In [32], the study aimed to determine the feasibility of conducting a randomized controlled trial to assess the effectiveness of a humanoid robot (i.e., Kaspar) in assisting the development of social skills in children with ASD. The findings suggest that providing early intervention, such as the use of Kaspar, has the potential to improve children’s social skills by, for example, increasing joint attention, engagement, reciprocal communication, speech and language therapy, and long-term outcomes.

#### 4.2.2. Experiment Period

This detail indicates the period needed to carry out the experiment in months. As shown in Table 3, below, the majority of the studies conducted their experiments in less than one month.

For example, Arshad et al., in [30], examined the efficacy of robots as assistive technology learning tools for children with ASD. A robot was created and programmed using LEGO Mindstorms EV3 to teach the fundamental concept of place value in mathematics. The children were assessed at the end of each session after participating in both traditional and robotic intervention lessons in one day, and the findings indicate a better attitude toward learning. 

One study [16] described a novel music therapy platform based on robots for modeling and improving the social responses and behaviors of children with ASD. The findings suggest that the proposed robot-music-based therapy platform is an appealing and promising assistive tool for improving fine-motor-control and turn-taking skills in children with ASD.

**Table 3:** Classification by experiment duration in months.

Experiment Period (Month)	References
<1	[12,14,15,18,21,23,27,29,30]
1–2	[9,20,31]
3–5	[10,17,19,25]
>5	[13]
Undefined	[11,16,22,24,26,28,32]

#### 4.2.3. Number of Sessions

The number of sessions used ranges from 1 session to more than 30 sessions depending on the experiment duration and the number of tasks and activities. Table 4, below, summarizes the article classification by the number of sessions.

For instance, Campos Panceri et al. in [23] introduce a new socially assistive robot used in psychosocial and cognitive therapy for children with ASD, using serious games such as Force Hammer; Music Therapy; Let Us Dance!; What is the card?; and Animal Detective. The experiments were conducted in just one session. After the experiments were analyzed, in comparison to static games, serious games developed for the robot were found to be an effective tool for recreational therapy because they provide greater mind–body interaction and promote greater therapy. 

On the other hand, the authors in [20] conducted a total of 194 sessions in 49 days, with 4 sessions each day. The study examined the sociopsychological and physiological effects of a parrot-inspired robot, KiliRo, which was developed to aid in the therapeutic settings of children with ASD. Using social network analysis, the researchers investigated the frequency of participants’ interactions with one another and assessed any changes in their interaction.

**Table 4:** Classification by number of sessions.

Number of Sessions (Total)	References
1–10 sessions	[9,10,12,13,14,15,18,21,23,24,30,31]
11–30 sessions	[19,22,25,27,28,32]
>30 sessions	[20,29]
Undefined	[11,16,17,26]

#### 4.2.4. Number of Participants with ASD and Age Groups

The number of children with ASD who participated in the experiments varied from 1 participant to 45 participants (mean = 18, median = 15, SD = 14, and variance = 204). For example, Santos et al. in [22] have only two ASD children who participated in their experiment. Two key contributions of this study are using triadic interactions (adult, robot, and child) with robotic mirroring and providing quantitative performance indicators. This study mainly proposes a robotic coaching platform for developing social, physical, and cognitive skills.

**Figure 3:**
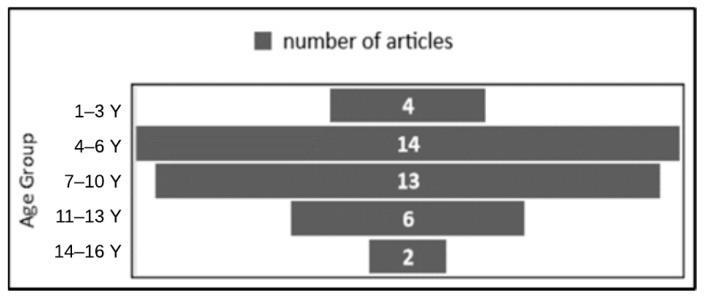
The age group of participants in the articles.

On the other hand, Soares et al. [29] had the largest number of participants, 45 children with ASD. The study investigated the impact of a humanoid robot on the development of socioemotional skills in children with ASD. The performance of children in game scenarios aimed at developing facial-expression-recognition skills is presented. Furthermore, participants in the articles ranged in age from 2 to 16 years old (min = 2, max = 16, and average = 8.5), as shown in Figure 3.

Several studies included children of various ages. For example, studies [11,29,31] had participants aged 4–6 years, and some other studies [18,24] carried out the experiment with children aged 11–13 years. To sum up, Table 5 and Table 6 present a summary of the classification by the number of ASD participants and by the age group in years of the participants with ASD, respectively.

#### 4.2.5. Evaluation Techniques

Robots have been developed for several interventions for ASDs. The evaluations used are varied as well, from conducting qualitative assessments such as interviews and surveys to carrying out quantitative assessments, such as a statistical analysis, in addition to predefined assessments such as the Gilliam Autism Rating Scale (GARS) and Goal Attainment Scale (GAS). Table 7, below, summarizes the article classification by evaluation techniques.

Qualitative assessment: This technique allows you to gain a thorough understanding of a program or process. It entails the “why” and “how” and allows for a more in-depth look at issues of interest and the exploration of nuances [33].

The authors of [19] investigated how the use of an educational robot as an assistive tool can help young children with ASD strengthen relationships and communicate with typically developing children. A class project was created that included all the children, including the child with ASD. 

An interdisciplinary road-safety program involving the construction, programming, and operation of a 3D LEGO robot bicycle model was implemented. A sociometric test was used for evaluation, and an interview was conducted to determine whether the child’s mother had noticed any differences in her child’s behaviors and road-safety awareness.

Quantitative assessment: This is an evaluation method that produces numerical indices. It seeks to provide precise answers to previously defined evaluation objectives as the final phase of the program evaluation [34]. For example, in [22], the study proposed a robotic coaching platform for developing social, motor, and cognitive skills. The researchers implemented two protocols, robot–master and adult–master, in which children performed various gestures guided by the robot or the adult, respectively, and then received feedback on movement execution. 

During the movement, the robot mirrors the subject in both protocols. The study also proposes new quantitative measures to evaluate the difficulty, repeatability, and mirroring of the movements selected for each protocol. These metrics could also be used to guide the development of new protocols in robotic mirroring coaching while also serving as potential metrics for continuous performance evaluation.

Predefined assessment: To give an idea, the authors in [24] aimed to describe a clinical experience with robot-assisted therapy (RAT) and its impact on the behaviors of a group of children with ASD in a therapeutic environment. At the beginning and end of each session, the parents and children were given satisfaction surveys about their behavior, enjoyment, and perception of the activity. Furthermore, the Vineland Social Maturity Scale was used to assess the impact of RAT on the core symptoms of ASD by considering the domains of communication, socialization, total score, and social age.

**Table 7:** Classification by evaluation techniques.

Evaluation Type	Number of Articles	References
Qualitative assessment	13	[11,14,18,19,21,24,25,26,27,28,30,31,32]
Quantitative assessment	10	[10,11,12,15,16,18,20,21,22,27,29]
Predefined assessment	12	[9,10,13,15,16,17,19,23,24,25,26,28,29,32]
Not defined	1	[12]

### 4.3. Research Challenges and Issues

To give researchers and practitioners a thorough understanding of the wide range of research possibilities in this field, we make an effort to discuss the challenges from a variety of angles, namely the robot level, algorithm level, experimental research level, and application level.

#### 4.3.1. Robot Level

Safety, security, and privacy: While robotics can greatly benefit children with ASD, there may be some risks or drawbacks that should be considered beforehand. One of the most important aspects that must be addressed is the ethical implications of using robots for children with ASD. For example, safety must be prioritized by ensuring that there is no danger of electrical shock and no sharp edges [35].

Researchers, specialists, and parents can retrieve information about the child’s performance and monitor the therapy’s quality. On the other hand, there are issues with information recording and storage: Who has access to the information? Are the data securely stored? To whom are the data allowed to be passed on? Hence, when collecting data, it is critical to consider all security and privacy concerns [14].

Robot–child interaction: There are several issues associated with child–robot interaction that must be addressed in order to properly complement current ASD techniques. Child–robot interaction may differ fundamentally from adult–robot interaction in that children are not simply miniature adults. Their neuro-physical, physical, and mental development differs, which may result in completely distinct circumstances [36].

**Figure 4:**
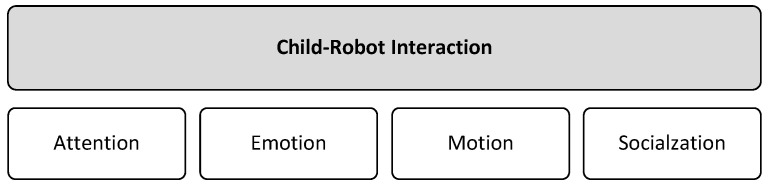
Child–robot interaction aspects.

We later discuss some challenges related to children with ASD and robot interaction based on four aspects that could influence learning interventions using robotics (as shown in Figure 4).

The ability of robots to inspire engagement and motivate children to participate in therapy sessions is the fundamental argument for using robots in behavioral therapies for children with ASD [9,37]. Even though a robot has the potential to capture a child with ASD’s attention and engagement, it is likely that its initial appeal will wear off over time, as the novelty effect of the new technology fades away [37]. Previous research acknowledges that there is a decrease in attention and engagement in child–robot interactions over time [9,37]. Thus, the challenge is how can we utilize robots in long-term interactions with children without causing them to lose interest, and without necessitating an impractical amount of content creation [13]?

Individuals with ASD have difficulty recognizing other people’s facial expressions, thus making interacting with others challenging [38]. Numerous studies have found that children with ASD develop slowly in terms of identifying and expressing facial emotions [13,17,38,39]. To overcome this gap, a variety of interventions have been developed to assist children in developing these skills, many of which use technical devices, such as robots [17], computers, and avatars, as social mediators [39].

While evidence indicates that these approaches were beneficial, some robots lack the ability to show human-like nonverbal behavior, which is essential for effective human–robot interaction [15]. In such circumstances, robot-specific nonverbal behavior, such as changing the color of the eyes to convey emotional meaning, is used [15]. However, it is unknown whether or not it is effective [40]. Furthermore, there has been limited systematic research on user facial expressions during human–robot interaction [41].

Children with ASD may show a wide range of challenging behaviors, including aggression toward others, disengagement, tantrums, property destruction, and meltdowns [42]. Challenging behaviors, which are frequently associated with autism spectrum disorders, can be harmful to both the children and the people around them. Throwing objects at people, kicking objects, hurting oneself, or banging on objects, for example [43], might be dangerous. Previous research has shown that children may be aggressive toward robots. During the interaction, some aggressive behaviors may lead to harmful scenarios for the robot [42].

Children’s worries and expectations about technology have a significant impact on its growth, impact, and social acceptance [44]. Despite research into children’s perceptions of social robots, there is a need to better understand how children’s hopes and fears influence their perspectives of the future [44]. Not all children may gain equally from robot interventions, and the advantages may be decided by children’s views toward robots and social deficits [45]. Specialists noted that it is possible that not all children like robots, and that some of them may even have an aversion to them [12].

#### 4.3.2. Algorithm Level

Several studies have been conducted to investigate the use of robots equipped with AI and machine-learning technologies in a variety of educational contexts. Hence, integrating these technologies into the learning environment would likely lead to superior learning outcomes [22].

Currently, most assessment and evaluation processes in the field of education are conducted manually, using traditional methods [11,14,18,19,21,24,25,26,27,28,30,31,32], which can be time-consuming and difficult to analyze. Furthermore, there is a lack of integration of AI and machine learning in such circumstances. Therefore, there is a need for further research to explore the potential benefits of integrating AI and machine learning into the assessment and evaluation process in education.

Furthermore, one of the most significant challenges is the lack of an open-access and large image dataset, which is required for developing machine-learning image-classification models. To train machine-learning models, researchers commonly need to use huge, diverse datasets that include the faces of ASD children. However, due to data privacy concerns, this may be challenging [18,24,30].

#### 4.3.3. Experimental Research Level

Research in the field of education and learning has predominantly focused on short-term experiments, lasting less than a month and with limited sessions. For example, Arshad et al. in [30] examined the efficacy of robots as assistive technology learning tools for children with ASD. The children were assessed at the end of each session after participating in both traditional and robotic intervention lessons in one day. In addition, the number of sessions conducted in most studies [9,10,12,13,14,15,18,21,23,24,30,31] ranges from 1 session to 10 sessions.

However, it is hard to assess learning progress accurately and effectively with short-term experiments. Additionally, the current body of research has been limited to experiments conducted with a small group of participants [9,19,25] and in specific environments. Therefore, there is a fundamental issue of generalization for all research approaches, as it is common for a child to perform better in a particular learning setting such as a therapy room but then struggle to apply the learned skills to different situations [12].

#### 4.3.4. Application Level

The use of robots in treatment has grown in popularity in recent years, but there is still a considerable obstacle to widespread implementation. The absence of therapists with a technological background, in particular, represents a substantial difficulty in efficiently using robots in therapy [32].

Many therapists may lack the essential skills and understanding to handle and communicate with these technological interventions, thus limiting the potential benefits of robot-assisted therapy. Another application-level challenge of using robots in therapy is the need for customization and personalization of robot interactions for each individual patient [32].

## 5. Discussion

**Figure 5:**
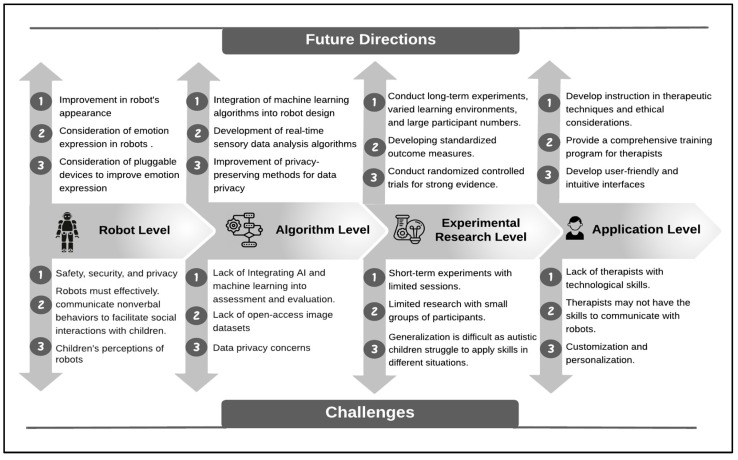
Challenges and future directions.

In order to fully realize the potential of robot-assisted autism therapy in terms of improving outcomes for children with ASD, it is imperative to address the opportunities and challenges associated with this field from a variety of angles. The levels of challenges that have been mentioned previously—robot level, algorithm level, experimental research level, and application level—must be addressed in future research. These challenges are mapped to the upcoming research directions in Figure 5, which provides a general overview of the possible research directions to be investigated.

Robot Level: The appearance of the robot is expected to influence user acceptance. Despite the encouraging impacts of robots on children with ASD during treatment sessions, not all robots appear to be equally suitable for this purpose. For example, Ref.[46] reported that children with ASD performed better during interaction experiments with doll-like robots with non-humanlike looks than during identical experimental sessions with the same robots with humanlike facial looks and that they avoided looking at the robot’s face when it had humanlike features.

In addition, not all robots can express emotions in order to improve human–robot interaction. For example, the NAO robot is a popular platform in the field of HRI. This robot primarily displays emotions through gestures and colored LED eyes, but it cannot express facial expressions due to its flat and inanimate white face [40]. The authors in [47] suggested a pluggable eyebrows device with two degrees of freedom separated in this work.

Algorithm Level: Machine learning and AI are rapidly advancing fields that may have significant potential in the field of autism treatment. One application of machine learning for autism treatment is the use of robots. Integrating machine-learning algorithms into the design of robots will create more customized and effective interventions for children with autism [22]. For example, in [48], the authors presented robots that perform essential roles and provide benefits in the interaction of autistic children. In the absence of a dialogue corpus, they collected and integrated conversation data for children with autism and presented it in order to use a neural network to build a robot dialogue system that generates answers freely and without restrictions, as well as design robot movements to attract the attention of children with autism.

On the other hand, it is critical that we find ways to use machine learning while ensuring data privacy. One of these solutions is to use privacy-preserving machine-learning techniques such as differential privacy, which can add noise to data to make it more difficult for a data breach to occur. Therefore, these methods could reduce data-sharing worries [49].

Experimental Research Level: Accurately assessing learning progress is an essential step in determining the effectiveness of educational programs [50]. To address this, there is an increasing demand for long-term experiments, a varied range of learning environments, and a large number of participants [9,12,25]. Using these approaches can improve learning assessment accuracy and promote higher generalization of learned skills. Researchers can obtain a deeper understanding of the factors that influence learning and develop more effective educational practices that can be applied in a variety of contexts by undertaking research in these areas.

Application Level: The use of robots in therapy has received a lot of attention in recent years as a potential way to improve the engagement and progress of children with ASD [15]. Robot intervention in therapy, on the other hand, demands extensive training for therapists in order to handle the sessions. This is because robots provide distinct challenges, such as programming and maintenance, as well as the need for therapists to effectively interact with the technology.

Therapists must be instructed in the use of appropriate therapeutic techniques, as well as the ethical considerations and possible consequences involved with the use of robots in therapy [32]. As a result, a comprehensive training program is recommended to ensure the proper integration of robots in therapy and gain the full benefits of this technology.

## 6. Conclusions

Several studies have demonstrated that technology can facilitate clinical assessment, diagnosis, and intervention in the area of ASD [51,52,53,54]. Several interventions for children with ASD have been developed with the goals of improving cognitive ability and daily living skills, increasing their ability to interact and engage in the community, and trying to reduce symptoms. For instance, Frolli et al. [54] explored the use of virtual reality to support social skills in children with ASD, while Marocco et al. in [53] investigated the use of technology to identify children with ASD through motor abnormalities. Moreover, video modeling has shown promise in promoting the development of emotional skills in children with ASD, as demonstrated by Regaa et al. in [52]. Overall, these studies highlight the potential of different technologies in providing a comfortable and engaging environment for individuals with ASD to improve outcomes in clinical assessment and intervention.

The impact of robotics on children is critical, as robots can be used to aid in their development and intellectual growth. Accordingly, more emphasis must be placed on how educational robots can be better integrated into the lives of children. With the constant development of technology, it is worthwhile to comprehend the potential of robots as effective learning aids. This study examined 24 published articles on the use of robots in educational and learning environments.

This review assisted us in better understanding the various robot intervention techniques for people with autism in the field of education and skill development. Hence, it could be regarded as a reference source for robotics researchers, academics, and practitioners. According to the results of this review, it is suggested that therapists, children with ASD, and parents consider using robots as an assistant to increase their acceptance, create more trust, and improve the quality of learning sessions.

The results of this review suggest that robots such as NAO and Kaspar may be useful tools for increasing acceptance, creating trust, and enhancing the quality of learning sessions in autism therapy. However, further research efforts need to focus on identifying which specific skills and types of interventions the robots are most effective for. Our review revealed that the optimal duration of the interventions and the long-term effects of using these robots in therapy are still unclear and require further investigation.

Additionally, the number of sessions and the frequency of the interventions may play an important role in their effectiveness, and further research is needed to explore these factors. The small sample size and age range of participants in the studies included in our systematic review suggest that robot-assisted interventions have been primarily evaluated in small groups of children within a specific age range. While early intervention is critical in improving outcomes for individuals with autism, the optimal number and age range of participants for these interventions are still unclear and require further investigation. Therefore, more diverse and representative samples of individuals with autism need to be included in future research studies to ensure the generalizability of the findings.

In conclusion, this study identified and emphasized the research issues and challenges that need to be addressed by researchers in the field of robot education and training. These issues have been classified into four levels: robot level, algorithm level, experimental research level, and application level. Furthermore, the paper provides an overview of the current state of the area and highlights possible future research directions. Addressing these issues and seeking new research avenues will be critical to fully achieving the potential benefits of robot-assisted therapy. As a result, this study offers crucial insights for researchers interested in using robots in therapy, and it can serve as a roadmap for future research in this field.

## Figures and Tables

**Table 2:** Article classification by type of robot.

Robot Type	Number of Articles	References
NAO	10	[9,10,13,14,15,17,18,21,22,25]
Kaspar	5	[11,16,26,31,32]
Other	9	[12,19,20,23,24,27,28,29,30]

**Table 5:** Classification by number of ASD participants.

Number of ASD Participants	References
2–10	[9,12,13,17,18,22,24,25,27,30,32]
11–20	[11,15,28]
21–30	[10,12,19,20,21]
>30	[29,31]
Undefined	[16,26]

**Table 6:** Classification by age group in years of ASD participants.

The Age Group of ASD Participants (Years)	References
1–3	[9,11,14,15]
4–6	[9,10,11,12,14,15,17,20,21,22,23,25,27,29,31,32]
7–10	[9,14,17,18,19,20,21,23,24,27,29,30,32]
11–13	[13,14,18,24,27,30]
>13	[20,27]
Undefined	[12,16,26,28,32]

## Data Availability

Not applicable.
